# Redefining the “carrier” state for foot-and-mouth disease from the dynamics of virus persistence in endemically affected cattle populations

**DOI:** 10.1038/srep29059

**Published:** 2016-07-06

**Authors:** Barend M. deC. Bronsvoort, Ian G. Handel, Charles K. Nfon, Karl-Johan Sørensen, Viviana Malirat, Ingrid Bergmann, Vincent N. Tanya, Kenton L. Morgan

**Affiliations:** 1 The Roslin Institute at The Royal (Dick) School of Veterinary Studies, University of Edinburgh, East Bush, Midlothian, EH25 9RG, UK; 2 National Centre for Foreign Animal Disease, Winnipeg, Manitoba, Canada; 3 Danish Veterinary Institute for Virus Research, Kalvehave, Denmark; 4 Pan-American Foot-and-Mouth Disease Center (PAHO/WHO), Rio de Janeiro, Brazil; 5 Centro de Virología Animal, Instituto de Ciencia y Tecnología Dr. César Milstein, Consejo Nacional de Investigaciones Científicas y Técnicas (CONICET) Buenos Aires, Argentina; 6 Cameroon Academy of Science, Yaounde, Cameroon; 7 Institute of Ageing and Chronic Disease, School of Veterinary Science, University of Liverpool, Leahurst Campus, Neston, Wirral, CH64 7TE, UK

## Abstract

The foot-and-mouth disease virus (FMDV) “carrier” state was defined by van Bekkum in 1959. It was based on the recovery of infectious virus 28 days or more post infection and has been a useful construct for experimental studies. Using historic data from 1,107 cattle, collected as part of a population based study of endemic FMD in 2000, we developed a mixed effects logistic regression model to predict the probability of recovering viable FMDV by probang and culture, conditional on the animal’s age and time since last reported outbreak. We constructed a second set of models to predict the probability of an animal being probang positive given its antibody response in three common non-structural protein (NSP) ELISAs and its age. We argue that, in natural ecological settings, the current definition of a ”carrier” fails to capture the dynamics of either persistence of the virus (as measured by recovery using probangs) or the uncertainty in transmission from such animals that the term implies. In these respects it is not particularly useful. We therefore propose the first predictive statistical models for identifying persistently infected cattle in an endemic setting that captures some of the dynamics of the probability of persistence. Furthermore, we provide a set of predictive tools to use alongside NSP ELISAs to help target persistently infected cattle.

Foot-and-mouth disease (FMD) is a highly contagious viral disease (Picornaviridae, genus Aphthovirus) of even-toed ungulates (Artiodactyla) and is one of the most important economic diseases of livestock in the world. Following the success of the rinderpest eradication programme, a resolution adopted at the World Organization for Animal Health (OIE) and the Food and Agriculture Organisation (FAO) Global Conference on Foot and Mouth Disease held in Asunción, Paraguay in June 2009, tasked these organisations to work together on a programme for global control of FMD^1^. One of the central concerns for FMD control has been the possibility that persistently infected animals, often referred to as “carriers”, could trigger new outbreaks weeks or months after the disease has apparently been controlled^2^,^3^,^4^. Over 50 years ago van Bekkum^5^ defined FMD “carriers” as “animals from which virus can be recovered more than 28 days post infection”. The duration of this “carrier” state has been reported to last for varying periods for different species. These are widely quoted as up to 9 months in small ruminants, 3.5 years in cattle and 5 years in Cape buffalo^6^.

There are reports of transmission from “carriers” to susceptible animals. The strength of evidence for this is variable. Some of the earliest anecdotal evidence comes from Australia in 1871–72, where the last outbreaks there may have been due to imported “carrier” from the United Kingdom (quoted by Hedger^7^). Some of the strongest evidence comes from buffalo to cattle transmission both experimentally^8^ and under “natural” conditions^9^ in Africa, although transmission from sub-clinical acute cases or indirectly from people cannot be ruled out. High resolution molecular data to support the conclusions is missing. The role of buffalo is likely to be extremely limited outside Southern and Eastern Africa due to restricted wildlife population sizes^1^. It may only be important once eradication has been achieved in the cattle population. However, in spite of many experimental attempts, transmission from “carrier” cattle to susceptible cattle has not been achieved (as reviewed by Tenzin *et al*.^10^), although there is a report of transmission from a “carrier” cow to a pig at the Plumb Island Animal Disease Centre^10^. From the evidence to date, if “carrier” cattle do transmit virus, it seems to be a rare event and the likelihood of transmission declines with time since infection^10^,^11^. However, the small number of experimental studies on which this is based involved only small numbers of animals. Thus the true importance of the “carrier” state remains controversial and debatable^11^,^12^ and the dynamics of the decay in recovering virus post infection remains poorly quantified. This is particularly the case for transmission from naturally infected animals.

The anatomical site(s) of viral persistence are becoming clearer. Early studies suggested that the main site of persistence was the epithelium of the dorsal surface of the soft palate^13^,^14^, but later work suggested that the virus may survive in the germinal centres of the tonsils^15^. The most recent study, that looked at 28 anatomical sites, found very high prevalences of virus in the dorsal nasopharynx and dorsal soft palate^16^. Experimental studies of FMDV have detected virus in the oropharynx of up to 50% of cattle 28 days post infection, suggesting viral persistence maybe a common sequel to infection^17^. The relationship between vaccination and the “carrier” state is unclear. Viral persistence has been demonstrated in vaccinated animals^18^. Some studies have suggested “carrier” rates are lower in vaccinated populations^11^,^19^ whereas others found vaccination had little effect on the rate of development of the “carrier” state^20^. The precise mechanism of persistence, however, remains poorly understood^16^.

There have been very few attempts to study the prevalence and dynamics of the “carrier” state at a population level in naturally infected populations eg. Anderson *et al*.^19^, and Rashtibaf *et al*.^21^. Published information on FMDV implies that there are essentially 2 states at 28 dpi; “carriers” or “non-carriers”, based on whether or not live virus was cultured from a sample collected from the oropharynx using a probang cup. There is little or no information about the factors associated with virus recovery, the rates of decline in recovery (or clearance from an animal), or factors affecting the likelihood of viral recovery by probang.

Here we report the probability of recovering FMD virus in individual animals from a detailed cross sectional epidemiological study of FMD in Cameroon where infection with serotypes A, O and SAT2 is endemic^22^. We focus on the duration of viral persistence and the animal-level factors associated with it. We propose a redefinition of the “carrier” state as a dynamic state described by a probability function of persistence rather than an absolute binary state defined by an arbitrary 28 day cut off. We also develop a set of predictive models to use cheap non-structural protein ELISA tests to help target high risk animals in the field.

## Results

### Predictors of virus recovery by probang

**Figure 1:**
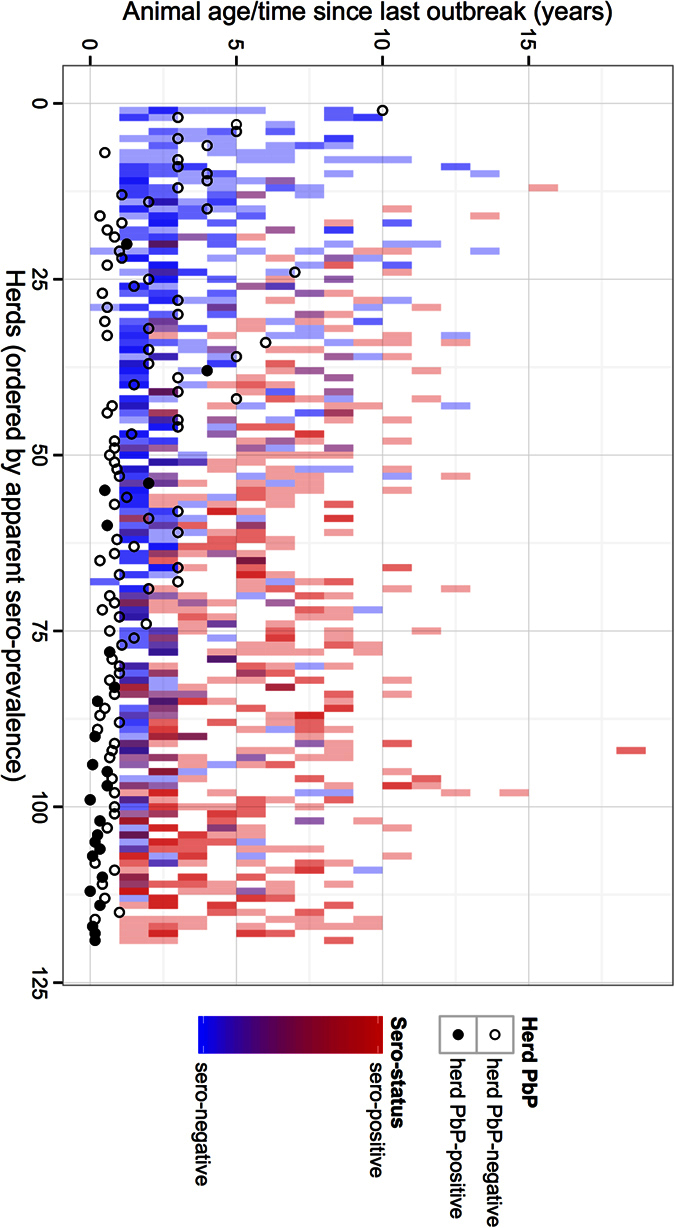
Tile plot showing the individual animal (n = 1107) serological status (based on the C-ELISA that is panspecific) by age (years) on the y-axis. Animals from the same herd are grouped in the same column and herds are ranked from highest seroprevalence to lowest along the x-axis. The circle then reports the probang status of the herd (solid circle if ≥1 were PbP) and is plotted on the y axis against the time in years since the last reported outbreak by the herdsman.

The final dataset consisted of 1,107 cattle with complete observations, after removal of animals with missing data for the variables of interest. These were mainly the first 30 herds in the original study where probangs were not collected. Thirty-eight animals were probang positive (PbP) with 26\38 ≤ 2 years old, 35\38 were in herds reporting an outbreak of clinical FMD in the last 12 months and 4\38 were in herds showing clinical signs on the day of examination. The serological and PbP status of individual animals are shown in Fig. 1. Most of the PbP animals were in herds with high seroprevalence in which herdsmen reported recent outbreaks. In general as the months since the last reported outbreak get greater, increasing numbers of seronegative juvenile animals (blue) were observed consistent with what might be expected. There were a couple of PbP animals from herds reporting outbreaks more than 12 months ago but these herds have few seropositive animals based on the NSP test again suggesting absence of virus circulation. Interestingly, of the 239 calves born after the last reported outbreak in herds that had a PbP calf, none had seroconverted based on NSP suggesting there was no sub-clinical viral circulation (i.e. where there are red tiles below a solid black circle in Fig. 1) and supporting the view that transmission from “carriers” is a rare event.

**Figure 2:**
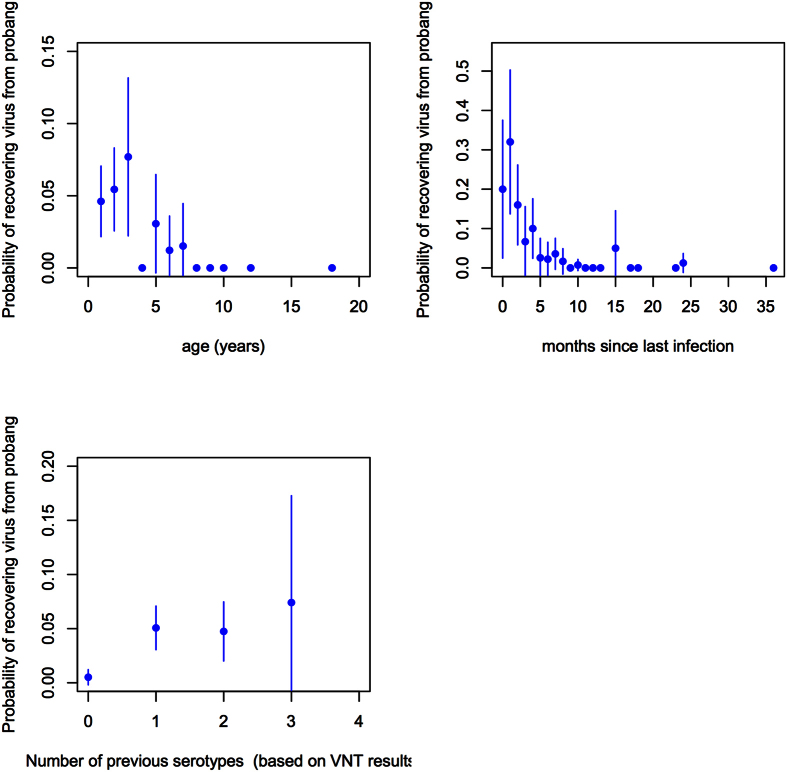
Univariable plots of the probability of an animal being probang positive (PbP) against (**a**) animal age (years), (**b**) months since last herdsman reported outbreak (months), and (**c**) the number of difference serotypes the animal was seropositive to based on serotype specific VNT results.

The main variables of interest, for which data were available, were animal age, months since last reported outbreak and the number of previous serotypes that the animal had been exposed to. The univariable relationships between these variables and PbP are given in Fig. 2. This shows a possible quadratic function between PbP and age and months since last outbreak. In contrast, the relationship between PbP and number of serotypes suggests that the number of previous exposures is not important so this was collapsed to a binary variable, *VNTany*, (seropositive by VNT to ≥1).

**Figure 3:**
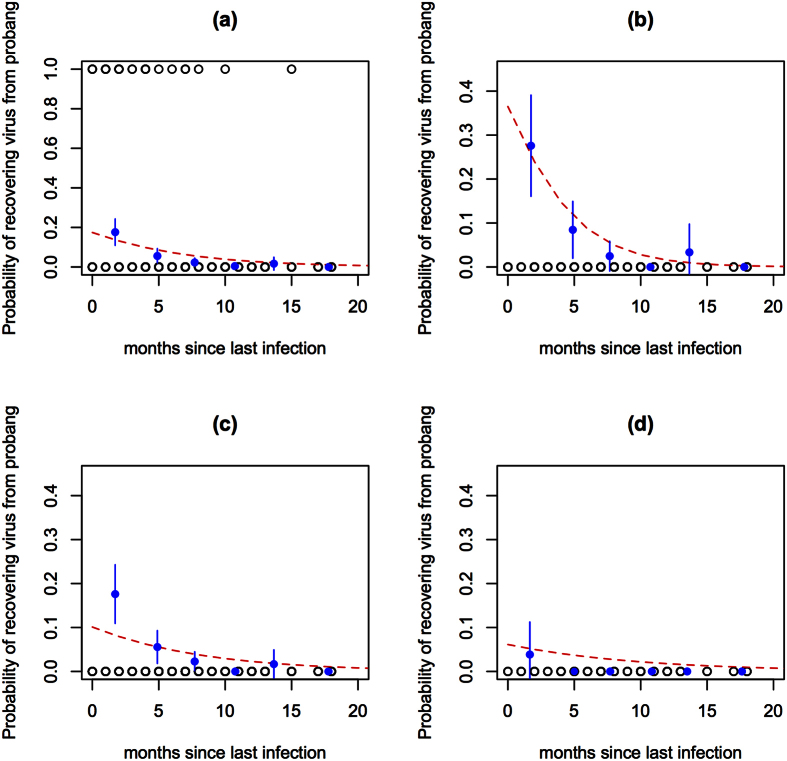
Plot of the model to predict probability (red line) of having a positive probang adjusted for months since the last herdsman reported outbreak and age (fitted as a quadratic) and the raw binned proportions to demonstrate the general suitability of he logistic model to the data. (**a**) Overall fit, (**b**) animals ≤2 years old, (**c**) animals >2 and ≤5 years old and (**d**) >5 years old.

The probability of being PbP was modelled using a standard multi-level logistic regression approach and allowing quadratic functions for the two continuous variables of interest, age of the animal (*age*) and the number of months since the last herdsman reported outbreak (*monlast*). Inclusion of additional variables was explored using a backward stepwise approach based on the model’s AIC. These included previous FMDV exposures to different serotypes as measured by VNTs (*VNTany*) and a categorical variable which represented the relationship between serotype of virus isolated by probang and the VNT serotype detected in that animal (i.e. did the animal have a detectable VNT response to the serotype of virus isolated from the probang). The final multi-level logistic model is presented in Table 1. The model diagnostics identified 3 covariate patterns with high leverage and influence but dropping each in turn did not result in any substantive change in the model parameters and so all observations remained in the final model. The overall fit and age specific fits are illustrated in Fig. 3 and suggest the model is a good fit supported by an AUC = 0.891 (0.838–0.945) and sensitivity of 0.82 and specificity of 0.86, using a cut-off of ≥0.06 for the probability of being PbP. At these relatively low apparent prevalences of PbP the positive predictive value of the model was only 17.5% while the negative predictive value was 99.2%

**Figure 4:**
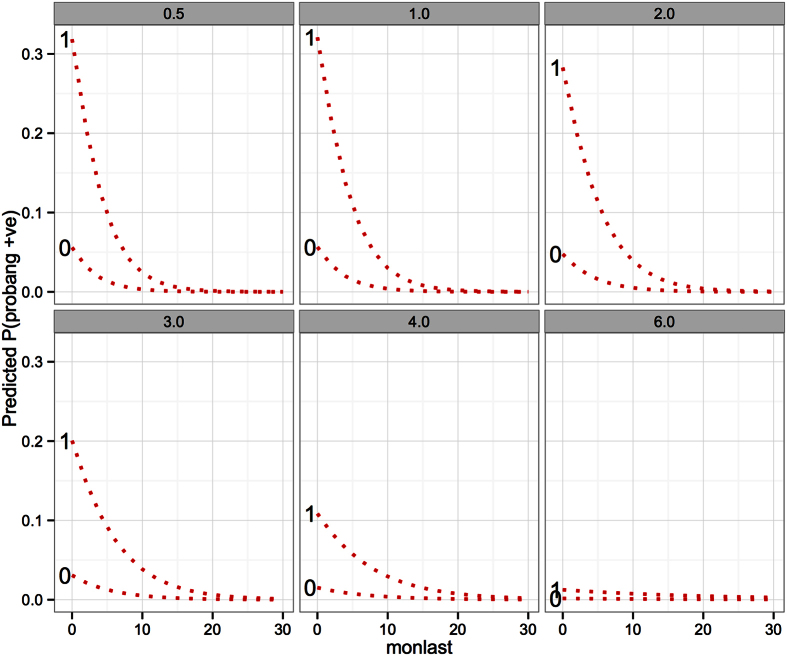
Model 1 predictions for a range of ages in years (line labels) and months since last FMD outbreak, stratified by number of previous serotype exposures as classified by the number of serotypes an animal was positive for based on the VNT result.

The predicted probabilities of being PbP for different ages, months since last outbreak and previous exposure (*VNTany*) are presented in Fig. 4 and show the marked decline in probability of recovering virus with time since the last outbreak. In addition, it shows clearly the declining probability of recovery of virus from older animals. Finally, these associations remain, but are modified by the number of previous serotype exposures, such that an animal with no previous exposure (as measured by the VNT) have a very low probability of virus recovery compared to animals with 1 or more previous serotype exposures.

### Predictors of PbP status when outbreak dates unknown

The second part of this analysis was to develop a set of predictive tools for use with different NSP ELISAs to help target which animals are likely to have recoverable virus, particularly if used in a surveillance setting when there is no information about outbreak dates, as in many endemic settings.

**Figure 5:**
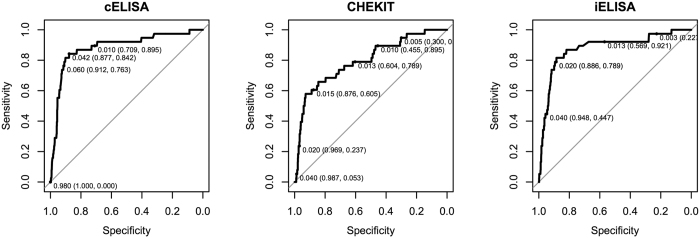
Receiver operating characteristic (ROC) curves for the 3 different NSP ELISAs models developed. The labels indicate different thresholds for the models and the resulting sensitivity and specificity in brackets

A standard logistic regression modelling approach was used to develop a series of three models using the continuous NSP serum antibody results from the cELISA, CHEKIT and iELISAs carried out on this same population, adjusting only for the age of the animal, on the assumption that little other information would be available. Age was best fit as the quadratic in all models. The summary of the parameter estimates and the model fit are given in Table 2. The receiver operating characteristic (ROC) curves for each predictive model are given in Fig. 5 and include exemplar threshold cut-offs for the models and the sensitivity and specificity estimates for that threshold value. In addition, we estimated the overall test sensitivity and specificity of the iELISA predictive model combined with the EITB, where only animals positive on both were considered positive. Interestingly this gave almost identical results to the predictive iELISA model on its own, thus the details were not included here. Overall the cELISA and iELISA appear to be very similar in fit and slightly better than the CHEKIT, although the 95% confidence intervals for the iELISA are very wide at the proposed threshold value.

## Discussion

The nature and role of the “carrier” state in the epidemiology of FMD has been the subject of debate for a number of years (eg. Kitching *et al*.^3^, Moonen *et al*.^6^, Sutmoller *et al*.^23^). Currently the definition of the “carrier” state in FMD originates from experimental infections and is defined as those animals in which the virus can be recovered 28 days after infection. This cut-off likely originates from an early pragmatic approach where isolation of FMD virus 4 weeks after infection was clearly concerning. However, this definition fails to capture the dynamics of the persistence and in our view has resulted in constrained thinking about the process and risks associated with persistence particularly in naturally infected settings. From a practical point of view two aspects of this are important; (1) the probability that an animal has viable FMD virus in its oropharynx (i.e. that it is persistently infected) and (2) the likelihood of transmission of this virus to susceptible in-contact animals, triggering a new outbreak. Both these components are needed to fulfil the definition of a “carrier” in infectious disease terms. Work by Orsel *et al*.^24^ and more recently by Charleston *et al*.^25^ suggests that the infectious period is relatively short. These experimental settings however may not be the most appropriate to understand the dynamics of persistence in cattle under natural field challenge and the variation in duration of virus persistence. The tendency to refer to “carriers” and “non-carriers” is therefore not particularly useful, and at worst, misleading given the lack of evidence for transmission between livestock species^10^.

This paper uses representative population based data from a field situation, in which multi-serotype FMD is endemic, to address the probability of recovery of viable FMDV via probang sampling, as a proxy and minimum estimate of the probability of an animal having virus and therefore being persistently infected. To our knowledge it is the first time this has been reported. In it we provide quantitative estimates of the probability of recovering viable virus by probang following naturally occurring clinical disease with various serotypes. We also identify the key factors affecting this to be time since last outbreak, age and previous exposure to FMDV (i.e. being seropositive to one or more serotypes). There was no effect of homologous or heterologous exposures to the serotype of the virus recovered by probang. The probability of being probang positive is represented by the equation:


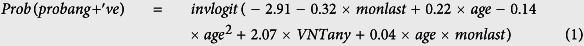


We consider this to be more biologically meaningful and practically relevant than a definition which uses a fixed time-bound cut off such as 28 days.

The relationship between virus recovery and time since the last outbreak is well recognized^26^ but incompletely characterized. Published data only refer to the upper limits of viral persistence in different species^6^. Our results suggest an extremely low probability, 3/422 (0.7%), of recovering virus from cattle more than 12 months after an outbreak. Interestingly, our study also indicates that the probability of viral persistence is influenced by age. This contrasts with previous reports which suggest that there is no age association with becoming a “carrier”^20^ and, by extension, the probability of recovering virus, although in subsequent studies these authors noted that in buffalo, 84% of viral isolates were recovered from animals 1–3 years old and the higher viral titres were from younger animals^27^.

This decreased probability of viral persistence with age is not associated with previous exposure and an adaptive immune response. When VNT status was added to the multivariable model, age remained a significant risk factor. Furthermore, being VNT positive appeared to increase the risk of being PbP. The only comparable studies might be where vaccinated animals are compared to non-vaccinated and here the results are conflicting, with some studies finding a decreased risk of developing the “carrier” status in vaccinated animals^19^ and others an increased risk^18^.

We used probangs for virus recovery and farmer reporting of the number of lunar months (moons) since the last outbreak. We have shown that herdsman reporting of FMD outbreaks in this setting is very reliable^28^ and probangs remain the standard technique for recovery of virus from the oropharynx, the proposed site of viral persistence. The examination of herds using probangs demonstrated that probangs, though not a perfect sampling system for virus recovery, may be more effective and reliable than collecting epithelial samples, where timely access to herds to collect epithelial samples is difficult. Also the whole post probang chain of freezing down the samples and then thawing and applying them to tissue cultures is also likely to be imperfect, however, this is poorly quantified. PCR has been proposed as the more sensitive approach^29^ but there does not appear to be any published evidence from field conditions estimating the sensitivity of either approaches.

It is generally accepted that probanging animals is insensitive in terms of identifying all the persistently infected animals in a herd at any given point and because we had no gold standard available we are unable to estimate the sensitivity of probanging. It can be difficult to collect a clean, rumen reflux free OP sample and the handling and shipping of samples is critical in order to minimize the decline in virus titres. We did record levels of rumen content contamination and found we were still recovering virus from heavily contaminated samples. We were able to carry liquid nitrogen to the field so that samples were diluted with buffer and frozen down within 1–2 hours of collection in most cases. Virus in the saliva may already be complexed with antibodies and will not be able to bind to the cell receptor and enter the cell to replicate and cause CPE in tissue culture. Treatment with chlorofluorocarbon can help displace the antibody^30^. In spite of these limitations of probanging generally, we were able to estimate the probability of recovering viable virus by probang from a randomly selected animal given its age and the time since the last outbreak in the herd, which though clearly related to the probability that the animal is persistently infected, is not the same thing but gives a minimum bound on persistence.

In some field situations where the disease is endemic it may be impossible to determine when the last outbreak of disease occurred. For this reason we investigated whether age and the level of serum antibody to each of the NSP ELISAs could be useful in predicting which animals were most likely to have viral persistence. Each of the models had the same form represented by the equation:


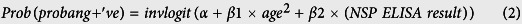


This relationship has also been suggested by others but has not be quantified before. Given that the NSP responses are not life long^31^ this may be a proxy for months since last infection. It is important to note that NSP tests^32^ or cytokines (with the possible exception of *TNF* − *α*^4^,^16^ are not particularly helpful to differentiate “carriers” directly but animals are unlikely to be persistently infected in the absence of an immune response as this analysis also supports. This can be helpful to potentially target higher risk animals following serosurveilance or identifying animals to probang to maximise chances of recovering viable virus and for sequencing.

This analysis has focused on the probability of virus recovery as this is what we were able to reliably measure. However, this clearly has important implications in improving our understanding of the related persistent status and, indirectly, also the “carrier” status. However, the classification of an animal as a “carrier” outside of experimental settings, it is not particularly useful, as it fails to capture the dynamic aspects of declining likelihood of recovering viable virus with time and age of animal. It also implies these animals are infectious. All the information to date suggests that the risk of transmission from persistently infected cattle to naive cattle is extremely low^10^. Although not the primary focus of this study, our results found no infected calves born after an outbreak in herds with persistently infected animals, which supports the experience from South America, where extensive field data has demonstrated the lack of evidence of transmission from “carriers” to sentinel unvaccinated young cattle or other susceptible species^11^.

In conclusion, there is still a lack of basic epidemiological data on how FMD persists at the population level in endemic, uncontrolled settings and what role “carriers” or, more usefully, persistently infected animals might have. This study has identified a strong relationship between both age and months since the last outbreak and the probability of recovering virus from probang sampling in endemic settings. These results directly impact our understanding of the “carrier” state in FMD and suggest that, rather than viral persistence being a simple binary condition triggered at some arbitrary time of 28 days post infection, we would be better to look at the dynamics of viral persistence and understand it from a probabilistic point of view where some animals are slower to clear the virus than others and the factors that influence persistence. The risk of transmission from these animals still needs to be established and we would argue that the continued use of the term “carrier” constraints thinking about these problems and the way we design our studies. What is reported in much of the body of FMD literature is actually about persistence and we should replace the term “carrier” with persistence and then look to understand the risk of transmission from persistently infected animals.

## Methods

### Study design and sampling

A cross-sectional study design was used and a stratified, two-stage random cluster sample of cattle herds in the Adamawa was selected. The study population has already been described in detail^33^. Briefly, the Adamawa Region of Cameroon lies between latitudes 6°N and 8°N and covers an area of about 64,000 km^2^. The final sample of 1,107 animals with complete data from 119 herds was drawn from the original sample of 1,377 animals from 147 herds across 54 veterinary centres which were visited between April and November 2000. A standardised questionnaire was used to collect herd-level information on the management of the herd, movements of animals and contacts with other herds and wildlife^33^. In addition the herdsmen were asked “how many months it had been since the last outbreak of FMD in the herd” to give an approximate measure of time since clinical disease was last observed in the herd. We have demonstrated from other analyses that herdsmen reporting is very reliable when compared against serology^28^.

A sample of 5 adult (>2 year old) and 5 juvenile (8–24 months old) cattle were randomly selected from each herd blinded to any information about previous clinical FMD signs in the animal (though occasionally there were fewer if the herds was small). The herdsman was asked for the age of each animal and the dentition was also recorded. A serum sample was taken from the jugular vein into a 10 ml vacutainer tube, allowed to clot and then separated using a 12-volt portable field centrifuge (Vulcon Technologies, Grandview, MO, USA). Sera were alliquoted into 2 × 1.8 ml cryovials (Nunc, Roskilde, Denmark) and kept at 4 °C in a portable gas refrigerator for up to 14 days until being frozen down at −20 °C. Samples were transfer between labs on dry ice. Oropharyngeal scraping/fluid (OP) was collected into a labelled 25 ml universal containing 2–4 ml of 0.08 M buffered phosphate with antibiotics^34^ using a probang cup^5^ and cryopreserved the same day. Cryopreserved samples were maintained in liquid nitrogen and shipped to the FMD-WRL in the United Kingdom where they were transferred to a −80 °C freezer prior to thawing and culture. Probang sampling was only started from the 32nd herd onwards explaining the reduced sample set available for this analysis.

A full description of the processing of probang samples is given by Bronsvoort *et al*.^35^. Briefly, 0.2 ml of probang suspension was inoculated onto each of 5 bovine thyroid cell (BTy) monolayers in flat sided culture tubes^36^. The cells were examined every 24 hours for cyptopathic effect (CPE) and any tubes showing CPE harvested. If no CPE was observed after 72 hours the sample was classed as ‘no virus detected’ (NVD). Due to resource constraints, because of the large sample size, the first passage was run for 72 rather than the standard 48 hours and a second passage was only done on samples showing non-typical CPE or where only one or two of the tubes showed CPE. The supernatant from such samples was filtered using a 2 *μm* micropore filter to remove bacterial contamination and re-inoculated onto 5 fresh Bty cultures. Supernatants from CPE positive BTy cultures were tested for the presence of FMDV antigen using the WRL indirect sandwich ELISA^37^,^38^.

The sera were screened by virus neutralisation test (VNT) for serotype specific antibodies to serotypes O, A and SAT2 based on the 3 serotypes isolated from this sample^35^ and the results are described elsewhere^22^. For the purposes of this analysis each animal was classified according to the number of serotypes it was positive for (based on the standard 10^1.64^ or 1:45 dilution cut-off) on an ordinal scale from 0 to 3 (ie. negative for all serotypes to positive to all three serotypes).

The sera were also screened using 3 pan-serotype non-structural protein (NSP) ELISAs: an indirect (I)-ELISA, the CHEKIT-ELISA and a competitive blocking (C)-ELISA. In addition, an Enzyme-linked immunotransfer blot assay (EITB) was used. Aliquots of the heat treated sera were sent to PANAFTOSA, Brazil, for screening using the I-ELISA^39^,^40^ (>9% classed as seropositive) and the EITB^41^ (which gives a binary result). The CHEKIT-3ABC-FMD ELISA (CHEKIT-ELISA) is described elsewhere^40^ and was carried out at the FMD-WRL (>30% classed as seropositive). The C-ELISA was performed as described^32^,^42^ and was conducted at the Danish Institute for Food and Veterinary Research in Kalvehave, Denmark (≤50% classed as seropositive).

### Statistical Analysis

Probang positive animals were defined as those from which FMD virus was recovered following culture. The original data were stored in an Access database (Microsoft). The R statistical software version 3.1.1 (http://cran.r-project.org/) was used to query the database, manipulate the data and produce exploratory plots. Univariable screening of putative factors was done in R using the standard *glm* function with a binomial link function. The linearity of continuous variables in the logit were checked using generalized additive models and the *gam* function in the *mgcv* package. A multivariable logistic regression model was developed using the *stepAIC* function allowing both forward and backward selection of variables including quadratic functions of age and months since last outbreak. The regression diagnostics Δ*D*, Pearson’s residual squared (Δ*χ*^2^) and influence (Δ*β*) were calculated and plotted against the predicted probability from the model as suggested by Hosmer and Lemeshow^43^ (not shown) using the the package *LogisticDx*^44^. Finally, a multi-level logistic regression model was estimated using the *nlme* package^45^ based on the variables remaining in the best fit standard logistic model but allowing for adjustment of the standard errors due to the design effects of cluster sampling within herds. The overall model fit was assessed using several tests: the area under the curve (AUC) of the receiver-operating characteristic (ROC) curves using the *roc* function in the *pROC* package^46^. Bootstrapped (n = 2000) 95% confidence intervals for the model sensitivity and specificity were estimated at the proposed thresholds using the *ci.se* and *ci.sp* functions in the *pROC* package.

## Additional Information

**How to cite this article**: Bronsvoort, B. M. d. *et al*. Redefining the “carrier” state for foot-and-mouth disease from the dynamics of virus persistence in endemically affected cattle populations. *Sci. Rep.* **6**, 29059; doi: 10.1038/srep29059 (2016).

## Figures and Tables

**Table 1:** Multi-level, multivariable logistic regression model (1) for being probang positive (PbP) based on animal age (years), farmer reported months since last outbreak (monlast) and whether the animals was positive for any of serotypes O, A or SAT2 by VNT (VNTany).

Variable	Coef	lci	uci	Odds Ratio	p-value
Intercept	−2.91	−4.89	−0.93	0.05	0.004
monlast	−0.32	−0.46	−0.17	0.73	<0.001
age	0.22	−0.61	1.06	1.25	0.448
age^2^	−0.14	−0.27	0.00	0.87	0.038
VNTany = 0	1				
VNTany = 1	2.07	0.46	3.67	7.90	0.016
monlast:age	0.04	0.02	0.07	1.05	<0.001
Herd (random effect)					
variance	0.5922				
intra cluster correlation	0.153				

**Table 2:** Summary table of three predictive models of probability of being PbP based on the different NSP ELISAs giving the parameter estimates and 95% confidence intervals.

Variable	cELISA	CHEKIT	iELISA
intercept	−0.97 (−1.87, −0.071)	−4.21 (−5.31, −3.11)	−4.21 (−5.11, −3.31)
age^2^ (months)	−0.072 (−0.108, −0.035)	−0.047 (−0.078, −0.016)	−0.057 (−0.091, −0.022)
Coef	−0.055 (−0/072, −0.037)	0.010 (0.000, 0.019)	0.025 (0.016, 0.033)
Model fit			
Threshold probability	0.042	0.015	0.019
Sensitivity	0.842 (0.622, 0.919)	0.684 (0.526, 0.816)	0.816 (0.658, 0.921)
Specificity	0.877 (0.639, 0.909)	0.797 (0.498, 0.939)	0.885 (0.292, 0.926)
AUC	0.884 (0.821, 0.948)	0.796 (0.717, 0.876)	0.877 (0.812, 0.943)

The predictive thresholds and model sensitivity, specificity and AUC are also given for each model.
